# Integrative transcriptomic and proteomic analysis reveals mechanisms of silica-induced pulmonary fibrosis in rats

**DOI:** 10.1186/s12890-021-01807-w

**Published:** 2022-01-07

**Authors:** Cunxiang Bo, Juan Zhang, Linlin Sai, Zhongjun Du, Gongchang Yu, Chao Li, Ming Li, Cheng Peng, Qiang Jia, Hua Shao

**Affiliations:** 1 Shandong Academy of Occupational Health and Occupational Medicine, Shandong First Medical University & Shandong Academy of Medical Sciences, Ji’nan, Shandong China; 2 Queensland Alliance for Environmental Health Sciences, The University of Queensland, Brisbane, QLD Australia

**Keywords:** Silicosis, Proteomics, Transcriptomics, SiO_2_, Rat

## Abstract

**Background:**

Silicosis is a systemic disease characterized by persistent inflammation and incurable pulmonary fibrosis. Although great effort has been made to understand the pathogenesis of the disease, molecular mechanism underlying silicosis is not fully elucidated. This study was aimed to explore proteomic and transcriptomic changes in rat model of silicosis.

**Methods:**

Twenty male Wistar rats were randomly divided into two groups with 10 rats in each group. Rats in the model group were intratracheally instilled with 50 mg/mL silicon dioxide (1 mL per rat) and rats in the control group were treated with 1.0 mL saline (1 mL per rat). Twenty-eight days later, transcriptomic analysis by microarray and tandem mass tags (TMT)-based proteomic analysis were performed to reveal the expression of mRNAs and proteins in lung tissues. Gene Ontology (GO) and Kyoto Encyclopedia of Genes and Genomes (KEGG) were applied to analyze the altered genes and proteins. The integrated analysis was performed between transcriptome and proteome. The data were further verified by RT-qPCR and parallel reaction monitoring (PRM).

**Results:**

In total, 1769 differentially expressed genes (DEGs) and 650 differentially expressed proteins (DEPs) were identified between the silicosis model and control groups. The integrated analysis showed 250 DEPs were correlated to the corresponding DEGs (cor-DEPs-DEGs), which were mainly enriched in phagosome, leukocyte transendothelial migration, complement and coagulation cascades and cellular adhesion molecule (CAM). These pathways are interrelated and converged at common points to produce an effect. GM2a, CHI3L1, LCN2 and GNAI1 are involved in the extracellular matrix (ECM) and inflammation contributing to fibrosis.

**Conclusion:**

Our comprehensive transcriptome and proteome data provide new insights into the mechanisms of silicosis and helpful information for more targeted prevention and treatment of silicosis.

**Supplementary Information:**

The online version contains supplementary material available at 10.1186/s12890-021-01807-w.

## Background

Silicosis is a systemic disease characterized by persistent inflammation and progressive pulmonary fibrosis. The disease is caused by long-term inhalation of occupational dust containing silicon dioxide [1]. Once onset, even if the exposure is terminated, silicosis can continue to develop and result in breath difficulty, low blood oxygen levels and eventual death [2, 3]. It is well established that the pathological process of silicosis involves phagocytosis, inflammation, extracellular matrix (ECM) remodeling, epithelial–mesenchymal transition (EMT) and fibrosis. However, the exact molecular mechanism underlying the silicosis is not fully elucidated and there are no specific drugs that can effectively alleviate or reverse silica-induced lung injury. Therefore, there is an urgent need to undersand the molecules and molecular pathways leading to silicosis.

Several distinct and complementary techniques have the potential to yield new perspectives on understanding the molecular activities and functions [4, 5]. Transcriptional profiling using high-throughput RNA sequencing technology is a useful method for identifying novel transcripts and analyzing gene expression changes in pathophysiology and toxicology [6, 7]. While gene expressions and their regulations are important in biological functions, protein expression represents outcomes of interaction between genetic and non-genetic factors and provides phenotype information of a disease [8]. Therefore, joint analysis of transcriptomic and proteomic data from the same samples can provide more comprehensive information on molecular mechanisms of a disease.

Silica particle-induced pulmonary fibrosis in rat model has been widely used in silicosis studies. In this study, we evaluated key target genes and proteins and associated pathways involved in silicosis through an integrative analysis with mRNA microarray and tandem mass tags (TMT) data. This comprehensive analysis of the transcriptome and proteome may substantially improve the overall understanding of the potential molecular mechanisms in silicosis.

##  Methods

###  Study protocol

This study was approved by the Committee on the Ethics of Animal Experiments of Shandong Academy of Occupational Health and Occupational Medicine (Protocol Number: 20190003). Twenty specific-pathogen-free (SPF) male Wistar rats (160–180 g) were purchased from Beijing Vital River Laboratory Animal Technology Co., Ltd. (Beijing, China), and housed in an SPF facility at 22 ± 2 C, 55% ± 10% relative humidity with a 12 h light/dark cycle and free access to water and chow. After one week, the rats were allocated to two groups with the random number table: the model group (n = 10) and the control group (n = 10), which were intratracheally instilled with 50 mg/mL silicon dioxide (1 mL per rat) and normal saline solution (1 mL per rat) respectively. Rats in the same group were housed together with five rats per cage. The individual rat was considered the experimental unit in this study. All rats were sacrificed on day 28 after administration. Please refer to our previous work for the detailed procedures of establishment and evaluation of rat silicosis model [9]. Based on the histological changes, three lungs from each group were selected for microarray and TMT assay to achieve the minimum requirements for biological replicates. All rats were intratracheally instilled with silicon dioxide under sodium pentobarbital anesthesia and sacrificed by carbon dioxide anesthesia after the exposure.

### Microarray assay

Total RNA was extracted using TRIzol Reagent (Cat# 15596-018, Life Technologies, Carlsbad, CA, USA) according to the manufacturer’s instructions and quantified by the NanoDrop ND-2000 (Thermo Scientific). The RNA integrity was assessed using Agilent Bioanalyzer 2100 (Agilent Technologies). An mRNA microarray analysis was conducted on each total RNA sample using a Low Input Quick Amp Labeling Kit, One-Color (Cat# 5190-2305, Agilent Technologies) and a Gene Expression Hybridization Kit (Cat# 5188-4242, Agilent Technologies) following the manufacturer's protocol. Slides were scanned using an Agilent Microarray Scanner (Cat# G2505C, Agilent Technologies) with the default settings. Feature Extraction software 10.7 (Agilent Technologies) was used to evaluate the raw datas which were normalized using the Quantile algorithm, Gene Spring Software 14.8 (Agilent Technologies).

### RT-qPCR assay

The lung tissues for RT-qPCR assay were same to the microarray assay. Total RNA was extracted as described above and double-strand cDNA was synthesized using the HiScript II Q RT SuperMix for qPCR (+gDNA wiper) Kit (R223-01, Vazyme, Nanjing, China) according to the manufacturer’s instructions. RT-qPCR was performed using ChamQ SYBR qPCR Master Mix Kit (Q311-03, Vazyme, Nanjing, China) on LightCycler^®^ 480 II Real-time PCR Instrument (Roche, Swiss). The primers were synthesized by Generay Biotech (Generay, PRC) based on the mRNA sequences obtained from the NCBI database (Table 1). The expression levels of mRNAs were normalized to ACTB and were calculated using the 2^−ΔΔCt^ method [10].

**Table 1:** Primer sequences

Gene	Forward primer	Reverse primer
*CHI3L1*	CCAATCACAGGGTCAGGATTA	TGAGGAAGTCGCATATCTCGTA
*LCN2*	CCGATGAACTGAAGGAGC	GTCGGTGGGAACAGAGAA
*GM2a*	TGTACGCCATGCTCCTTC	CCAGCTTGAACTTAAGATTCCA
*GNAI1*	GGTTCTGTGTTTGGCAGT	AGTCTGTGCAACGTTTATACAA

### TMT and PRM assay

Proteins from lung were extracted and digested, then the digested peptides were labeled using TMT Reagent-6plex Multiplex Kit according to the manufacturer's instructions (90066B, Thermo Scientific). LC–MS/MS was performed using a Q Exactive mass spectrometer (Thermo Scientific) combined with Easy nLC system 1200 (Thermo Scientific). The TMT labeling, peptide fractionation and mass spectrometer detection were carried out by Shanghai luming biological technology co., LTD (Shanghai, China). To examine the reliability of TMT mass spectrometry, four DEPs (GM2a, CHI3L1, LCN2 and GNAI1) were validated by parallel reaction monitoring (PRM) assay. Details of TMT and PRM assay were referred to our previous study [9].

### Bioinformatics analysis

Transcriptomics and proteomics data were first analyzed using Statistical Program for Social Sciences (SPSS) (SPSS Inc., version 20.0, United States). A fold change ≥ 2.0 and *P* values ≤ 0.05 was used to classify differentially expressed genes (DEGs). The significantly different expressed proteins (DEPs) were screened with a fold change ≥ 1.5 and *P* values ≤ 0.05 by t-test, and a multiple testing correction was performed using Benjamini and Hochberg procedure to control the False Discovery Rate (FDR), using *P* value (< 5%) [11]. Gene Ontology (GO) and Kyoto Encyclopedia of Genes and Genomes (KEGG) analysis were applied to determine the roles of these DEGs and DEPs, and the GO analysis was divided into biological process (BP), cellular component (CC) and molecular function (MF). *P* values less than 0.05 were considered significantly enriched.

### Correlation analysis of transcriptomics and proteomics

Genes are regulated at multiple levels during the expression process. At present, most studies have reported that the expression consistency between mRNAs and their corresponding proteins is not high enough. Therefore, we conducted a combined analysis of the proteome and transcriptome to gain a better understanding of the regulation of gene expression [12]. All expression data related to proteomics and transcriptomics were analyzed with the pearson correlation coefficient analysis between the model and control groups. The gene and its coding protein expressed with significant threshold of fold change and *P* value were identified and named as cor-DEPs-DEGs genes. The cor-DEPs-DEGs genes were further analyzed with GO and KEGG databases, and the spearman correlation coefficient was calculated.

## Results

### Histopathological evaluation of lung tissue

HE staining showed lung tissues were normal in control rats. In contrast, damaged alveolar structures, infiltrating inflammatory cells and silicotic nodule were observed in each rat from silica-treated group, which showed rat silicosis model was successfully replicated in this study. The detailed histological changes were referred to our previous study [9].

### DEGs and bioinformatics analysis

**Fig. 1:**
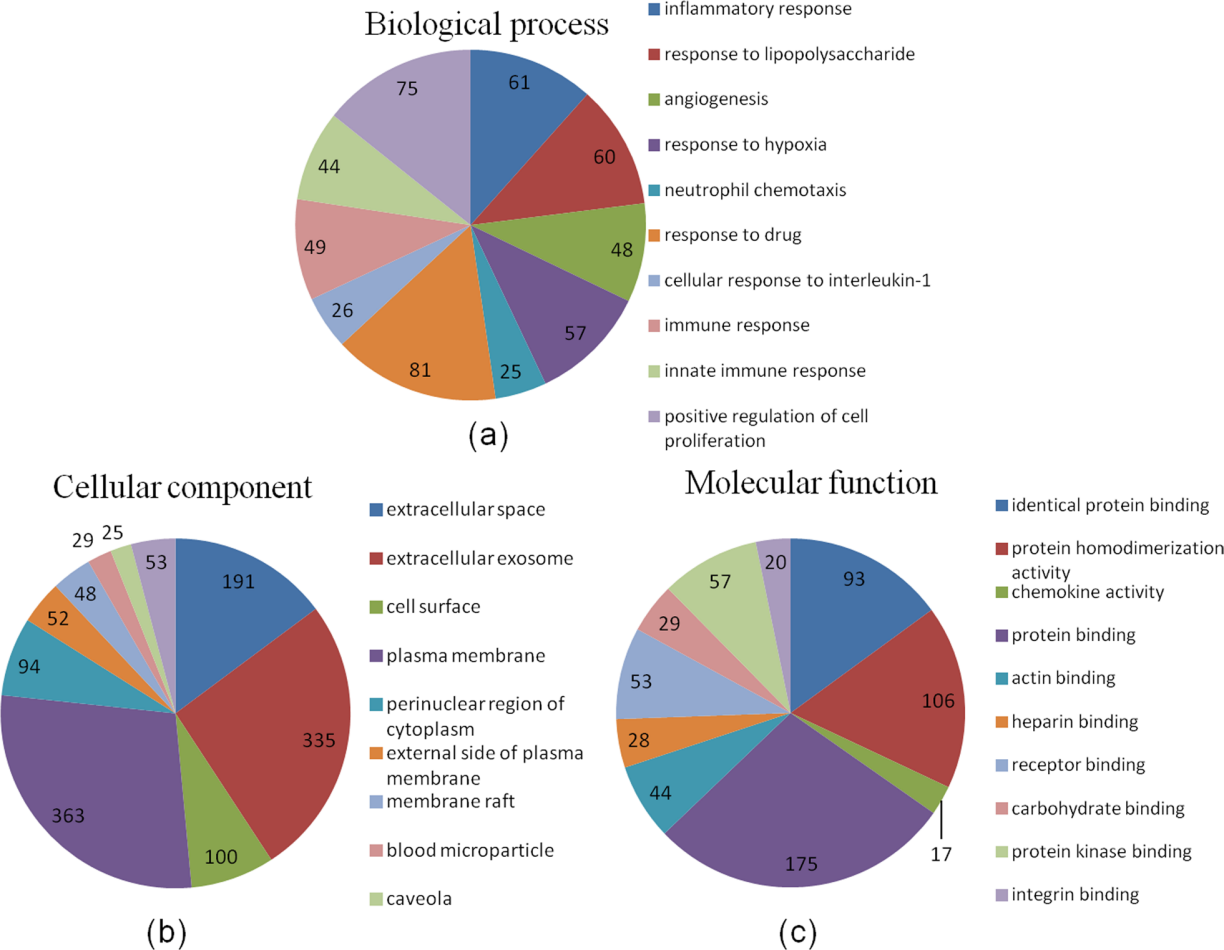
GO analysis of DEGs in lung tissues between the model and control groups. The top ten biological process categories (**a**), cellular component categories (**b**) and molecular function categories (**c**) are presented, FC > 2.0, *P* < 0.05

In total, 1769 DEGs were differentially expressed between the model and control groups. 952 DEGs were significantly upregulated, and 817 DEGs were significantly downregulated (Additional file 1: Table S1). These DEGs were enriched in 753 GO terms. The BP category was associated with inflammatory response, response to lipopolysaccharide and angiogenesis (Fig. 1a). The MF category was associated with extracellular space, extracellular exosome and cell surface (Fig. 1b). The CC category was associated with identical protein binding, protein homodimerization activity and chemokine activity (Fig. 1c). KEGG analysis proved these DEGs were enriched in 58 pathways, including complement and coagulation cascades, chemokine signaling pathway, cytokine–cytokine receptor interaction, Rap1 signaling pathway and Toll-like receptor signaling pathway (Table 2).

**Table 2:** The top ten KEGG pathways enrichment of DEGs in model rat lungs

Pathway ID	Description	*P* value
rno04610	Complement and coagulation cascades	8.26E−10
rno05150	Staphylococcus aureus infection	6.11E−08
rno05323	Rheumatoid arthritis	1.27E−07
rno04062	Chemokine signaling pathway	1.00E−06
rno04060	Cytokine–cytokine receptor interaction	1.78E−06
rno04015	Rap1 signaling pathway	5.94E−06
rno05133	Pertussis	1.02E−05
rno04620	Toll-like receptor signaling pathway	2.53E−05
rno05140	Leishmaniasis	2.90E−05
rno04142	Lysosome	5.16E−05

### DEPs and bioinformatics analysis

**Fig. 2:**
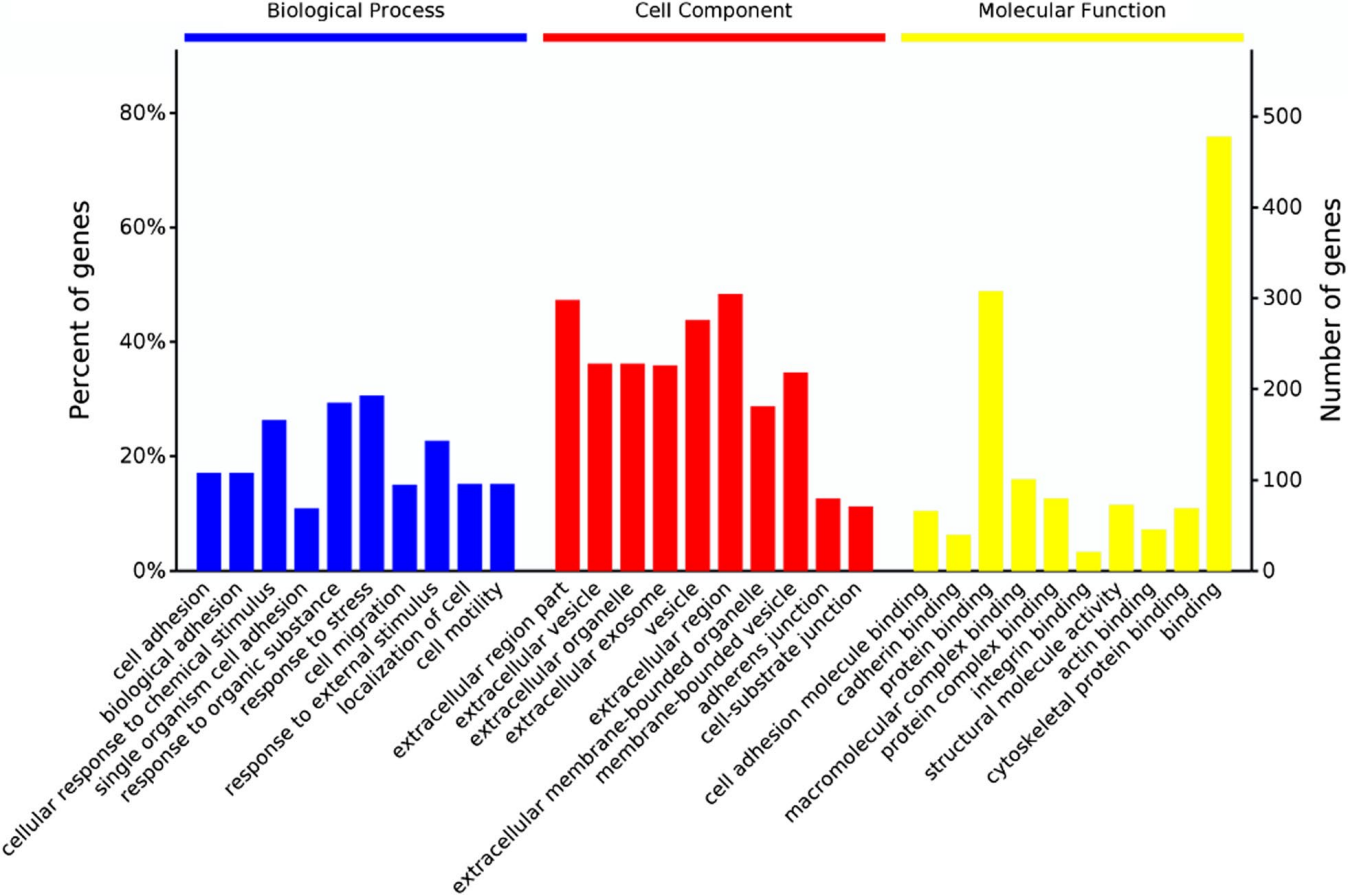
GO analysis of DEPs in lung tissues of rats from the model and control groups. The top ten biological process categories, cellular component categories and molecular function categories are presented, FC > 1.5, *P* < 0.05

A total of 650 DEPs were identified in rats from the model and control groups (Additional file 2: Table S2). Among them, 355 proteins were significantly upregulated, and 295 proteins were significantly downregulated. These DEPs were enriched in 1967 GO terms. The BP category was associated with cell adhesion, biological adhesion and cellular response to chemical stimulus. The MF category was associated with cell adhesion molecule binding, cadherin binding and protein binding. The CC category was associated with extracellular region part, extracellular vesicle and extracellular organelle (Fig. 2). KEGG analysis proved these DEPs were enriched in 40 pathways, including collecting duct acid secretion, ribosome, phagosome, lysosome, leukocyte transendothelial migration, glycosaminoglycan degradation, glutathione metabolism and ECM–receptor interaction (Table 3).

**Table 3:** The top ten KEGG pathways enrichment of DEPs in model rat lungs

Pathway ID	Description	*P* value
rno04966	Collecting duct acid secretion	5.74E−09
rno03010	Ribosome	3.56E−08
rno04145	Phagosome	4.78E−07
rno04142	Lysosome	8.09E−07
rno04670	Leukocyte transendothelial migration	3.18E−05
rno00531	Glycosaminoglycan degradation	4.01E−05
rno00480	Glutathione metabolism	0.000107
rno05323	Rheumatoid arthritis	0.000181
rno04614	Renin–angiotensin system	0.000495
rno04964	Proximal tubule bicarbonate reclamation	0.000584

### Protein-mRNA correlation analysis

**Fig. 3:**
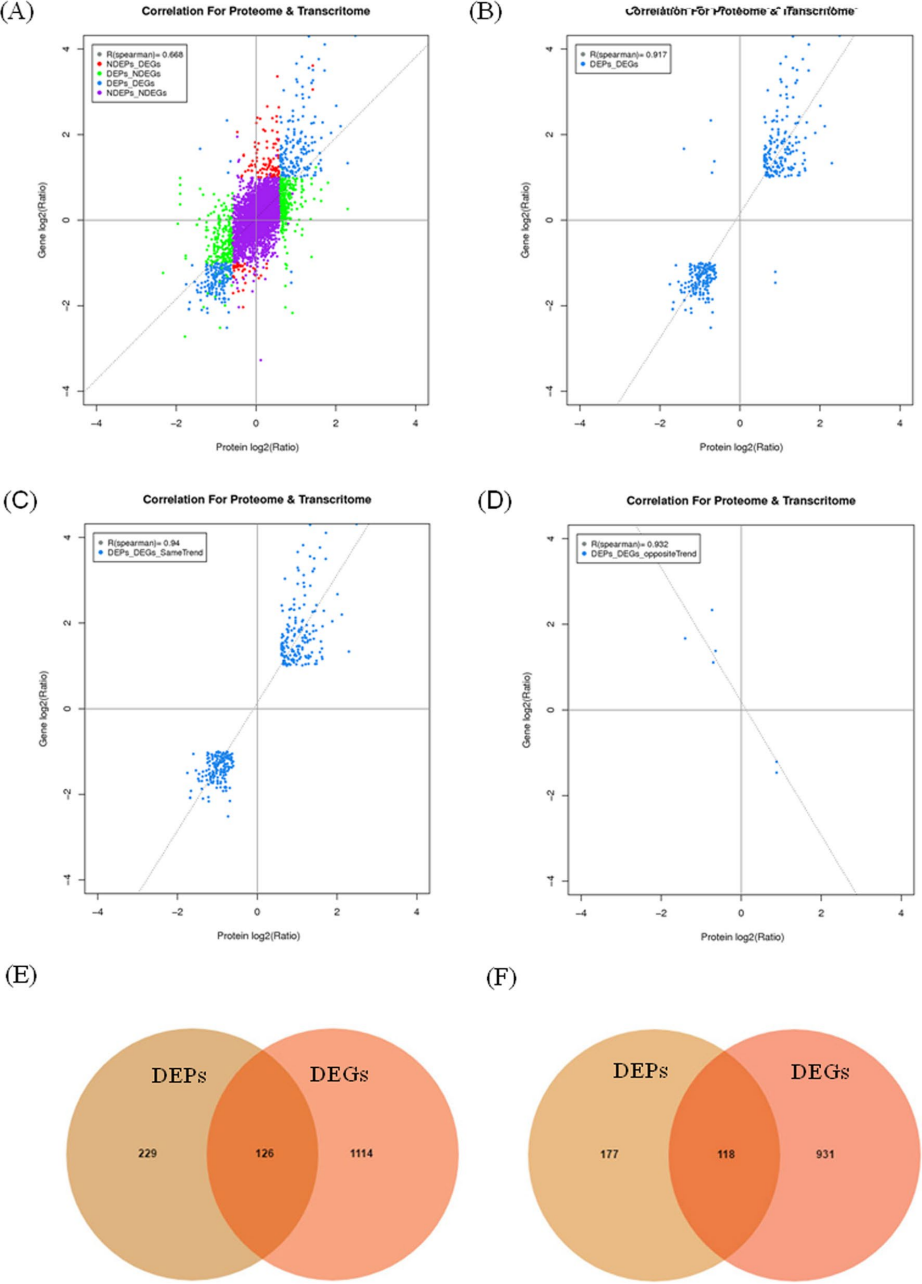
Correlations between the expression of proteins and genes. The horizontal-axis represents the protein expression level, and the vertical-axis represents the gene expression level. **A** Scatterplot of the relationship between genes identified in the transcriptome and proteome. **B** Scatterplot and correlation coefficients between DEGs and DEPs. **C** Scatterplot and correlation coefficients between DEGs and DEPs in the same trend. **D** Scatterplot and correlation coefficients between DEGs and DEPs in the opposite trend. **E** Venn diagram of upregulated DEGs and DEPs. **F** Venn diagram of downregulated DEGs and DEPs. Yellow part, DEGs; Red part, DEPs; Middle part, overlapped DEGs and DEPs

Integration analysis of above proteomic and transcriptomic data was conducted. All identified mRNAs with DEPs were matched, followed by transformation of DEPs and transcript volume ratios into log2 forms. An investigation of changes at both the transcript and tanslational levels revealed a relatively low correlation (Spearman correlation coefficient, R = 0.668, Fig. 3A) for all genes and proteins assessed. When we compared the 1769 DEGs with the 650 DEPs, the higher positive correlations were indicated (R = 0.917, Fig. 3B). When the correlation between DEGs and their corresponding DEPs with the same or opposite trend was analysed, higher positive and negative correlations were indicated (R = 0.940, Fig. 3C and R =  − 0.932, Fig. 3D). Among the cor-DEGs-DEPs genes, 126 upregulated (Fig. 3E) and 118 downregulated genes (Fig. 3F) were identified as the same trend as protein expressions, while 6 genes had the opposite trend with their correlating proteins (Additional file 3: Table S3). These cor-DEGs-DEPs genes might play important roles in lung injuries of rats exposed to silica particles [13].

**Fig. 4:**
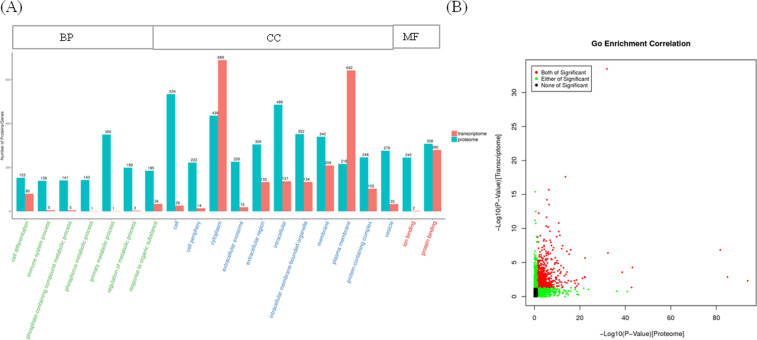
Correlation of GO enrichment between transcriptome and proteome. **A** Number of GO enrichment correlation between proteome and transcriptome. Each column in the figure represents a GO secondary annotation entry, Green represents DEPs, and red represents DEGs. The top twenty enriched terms of DEPs are presented. BP: biological process, CC: cellular component, MF: molecular function. **B** The overview scatter diagram of GO enrichment correlation between the protein level and transcript level of genes

**Fig. 5:**
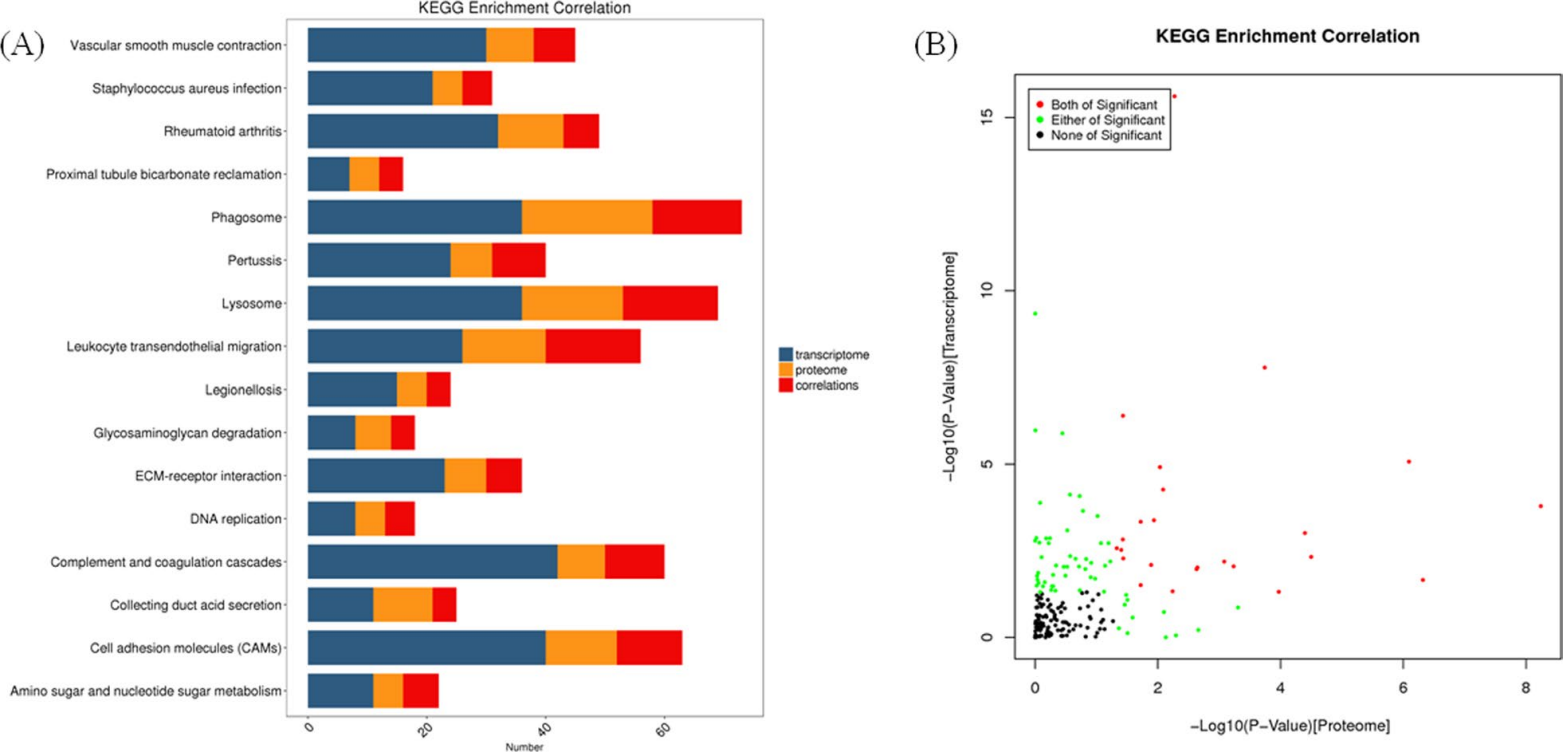
Correlation of KEGG enrichment between transcriptome and proteome. **A** Number of KEGG enrichment correlation between proteome and transcriptome. Each column in the figure represents a KEGG pathway, and different colors represent different histology. The yellow column in the figure represents the KEGG enrichment result of proteome, and the blue column represents the KEGG enrichment result of transcriptome. The red column in the figure represents the KEGG enrichment result of the correlated proteome and transcriptome. The ordinate is the name of the enriched KEGG pathway, and the abscissa represents the number of enriched proteomes and transcriptomes. **B** The overview scatter diagram of KEGG enrichment correlations between the transcript and protein levels of genes

To further explore the potential functions of those cor-DEGs-DEPs genes in silicosis, GO terms and KEGG pathways were enriched at the levels of transcriptome and proteome, respectively. GO terms were highly enriched at both mRNA and protein levels (Fig. 4 and Additional file 4: Table S4). The subcategory identified in the BP category were cell differentiation, immune system process and primary metabolic process. For CC category, intracellular, extracellular exosome and protein-containing complex were the most abundant categories. The most abundant MF were protein and ion binding. KEGG pathway analysis showed 16 pathways were highly enriched at both mRNA and protein levels, including phagosome, leukocyte transendothelial migration, complement and coagulation cascades and cell adhesion molecules (CAMs) (Fig. 5 and Additional file 5: Table S5).

### Data verification of microarray and TMT

**Fig. 6:**
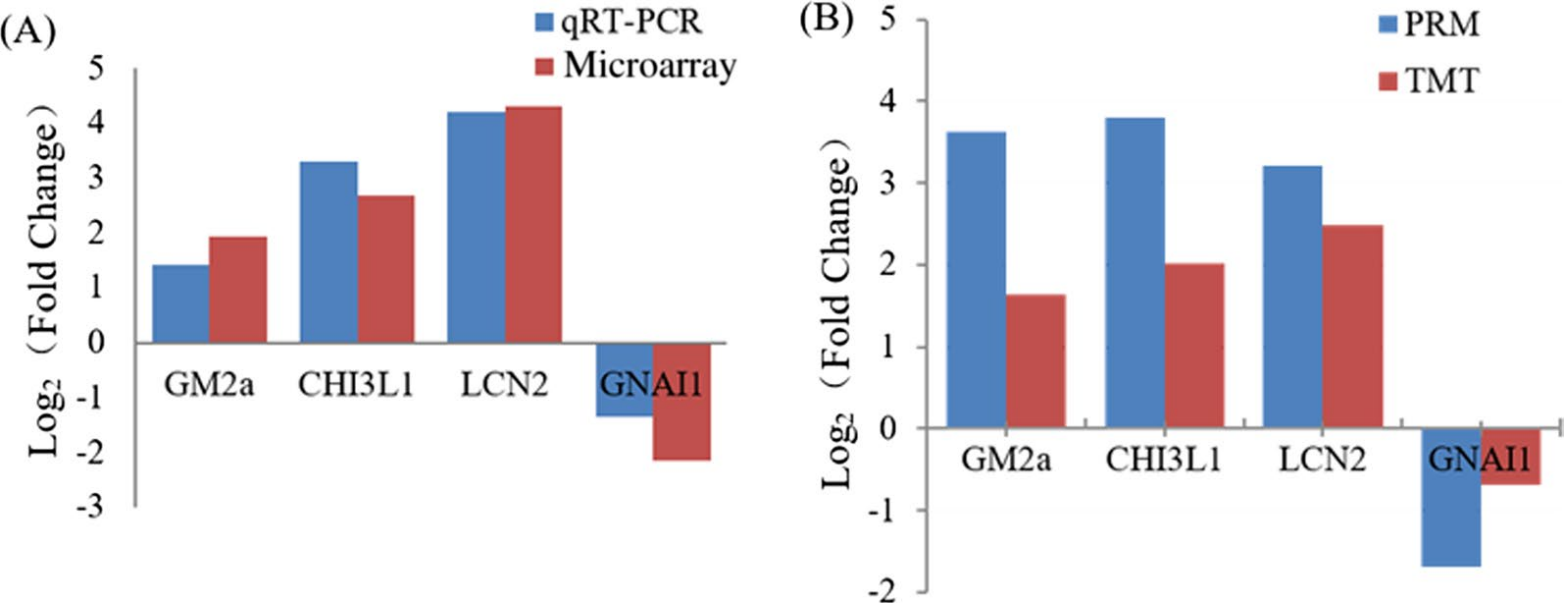
Data verified by RT-qPCR and PRM. **A** Comparison of the expressions of DEGs determined by microarray and qRT-PCR. log2 (Fold change) of the selected DEGs measured by microarray and qRT-PCR. GM2a: GM2 ganglioside activator; CHI3L1: Chitinase-3-like protein 1; LCN2: Neutrophil gelatinase-associated lipocalin; GNAI1: Guanine nucleotide-binding protein G(i) subunit alpha-1; The transcript expression levels of the selected genes were each normalized to that of the ACTB gene. **B** Comparison of the quantification results between TMT and PRM. log2 (Fold change) of the DEPS were measured by TMT and PRM

To validate the reliability of the microarray data in this study, we selected four DEGs (upregulated *GM2a, CHI3L1, LCN2* and downregulated *GNAI1*) for RT-qPCR assays. The results showed that all tested DEGs were significant changed between the model and control groups. The expression patterns were consistent with the results of microarray, although the folds of changes were different (Fig. 6A), which indicated that our transcriptome results might reflect the relative expression level of each gene in vivo. As shown in Fig. 6B, the three upregulated DEPs (GM2a, CHI3L1, LCN2) and downregulated GNAI1 were in the same trend as that observed when the protein levels were quantified by PRM, suggesting that the TMT data in this study was reliable. The difference of expression level (fold change) may be due to the different detection methods.

## Discussion

The aim of the study was to explore the molecular mechanism underlying silicosis and try to identify potential biomarkers that can predict the pathological progress of silicosis. To this end, we investigated the DEGs and DEPs in the lungs of the rats exposed to silica particles by microarray chip technology and TMT-coupled LC–MS/MS. The reliability of the results from microarray and TMT was confirmed by RT-qPCR and PRM assay. In total, 250 cor-DEPs-DEGs genes were identified in the lung tissues of rats from the silicosis model and control groups. Results of the KEGG pathway analysis indicated these cor-DEPs-DEGs genes were mainly enriched in phagosome, leukocyte transendothelial migration, complement and coagulation cascades and cellular adhesion molecule (CAM). For the first time, we found that cellular adhesion molecules (CAM), such as Gm2a, CHI3L1, LCN2, THBD, NADPH oxidase (NOXs) and vacuolar-ATPase are involved in pathogenesis of silicosis.

When the lung is injured, bleeding and leakage of plasma proteins from damaged surfaces in the alveolar space form a fibrinous deposit. Subsequently, the fibrin clot is invaded by proliferating fibroblasts, which forms the durable components of ECM [14]. Then the fibrin was degraded by plasmin. Decreased ECM degradation caused by deficient function of alveolar fibrinolysis plays an important role in the formation of pulmonary fibrosis in lung [15]. Excessive procoagulant and decreased fibrinolytic activities have been observed in animal models of lung fibrosis and pulmonary samples of patients with interstitial lung diseases [16, 17]. Procoagulant status in idiopathic pulmonary fibrosis (IPF) patients is associated with increased serum levels of factor VIII C, fibrinogen, homocysteine and protein C activity [18]. Thrombomodulin (THBD) plays an important role in anticoagulant by inducing plasminogen (PLG) to plasmin, which is inactivated by combining with C3b or CFH. Deletion of THBD can also cause abnormal activation of complement substitution pathway [19]. In this study we found that the downregulated THBD may result in the deficient of plasmin and ECM deposition. Fibrin and derivatives have been reported to exacerbate lung inflammation by stimulating the expression of leukocyte adhesion molecules, chemokines and proinflammatory cytokines [15]. Therefore, anticoagulant therapy could be helpful in silicosis treatment.

An imbalance of oxidation/antioxidation is a crucial mechanism of pulmonary fibrosis [20]. It has been revealed that the ROS generated by NADPH oxidase is important contributor to the pathological progression of fibrotic diseases in several organs, such as the heart, lung, liver and kidney [21, 22]. ROS accumulation can induce MAPK activation [20] and secretion of TGF-β further driving lung fibrogenesis [23]. NOX1, NOX2 and NOX4 play critical roles in initiating fibrosis through activation of hepatic stellate cells [24–26]. NOX2, the central phagocyte oxidase, locates in the plasma membrane and the membrane of intracellular vesicles of phagocytic leukocytes, such as neutrophilic granulocytes, eosinophilic granulocytes, monocytes and macrophages [27]. NOX2 needs p22^phox^ to form a stable heterodimer. During phagocytosis or at sites of inflammation, a complex of three cytosolic proteins p47^phox^, p67^phox^ and p40^phox^ moves to the NOX2/p22^phox^ to start the enzymatic activity of the oxidase. In the present study, lungs of rat model of silicosis showed increased phagocyte NADPH oxidase activity with upregulated five structural components NOX2 (*CYBB*), p22^phox^ (*CYBA*), p47^phox^ (*NCF1*), p67^phox^ (*NCF2*) and p40^phox^ (*NCF4*). ROS produced mainly by phagocytes NADPH oxidase plays an important role in silica-induced pulmonary fibrosis of rats. Highly specific NOX2 inhibitors may have potential to inhibit the development of silicosis.

The vacuolar-ATPase is an important pH regulatory complex using the energy produced by ATP hydrolysis to pump protons into the extracellular environment. The low extracellular pH may promote the degradation and remolding of ECM by increasing the activity of proteolytic enzyme such as matrix metalloproteinases (MMPs) and cathepsins [28, 29]. We found that the subunits of the vacuolar (v)-ATPase complex such as Atp6v0c, Atp6v1a, Atp6v1b2 and Atp6v1c1 were upregulated. Four cathepsins (CTSB, CTSD, CTSZ and CTSS) and MMP-9 were also upregulated. CTSS, one of the most potent elastases, can degrade fibrillar collagens, fibronectin and laminin. CTSB degrades collagen type IV, X and fibronectin [30]. CTSB/D can enhance expression of fibrogenic markers α-smooth muscle actin (α-SMA), TGF-β and Col1a1 in hepatic stellate cells (HSCs) from ASMase-null mice. Overexpression of CTSB was found to increase hepatic fibrosis in ASMase-null mice [31]. Low extracellular pH caused by enhanced V-ATPase activity could activate MMP-9 and cathepsins to promote the pulmonary fibrosis. In addition, the activity of MMP-9 is regulated by Lipocalin 2 (LCN2) by forming a macromolecular LCN2/MMP-9 complex, which protects MMP-9 from autodegradation [32, 33]. In particular, when bound to MMP-9, LCN2 may modulate various inflammatory mechanisms including acute phase proteins and the vascular repair process [34]. LCN2 and MMP-9 have been reported to participate in pulmonary fibrosis development [35].

**Fig. 7:**
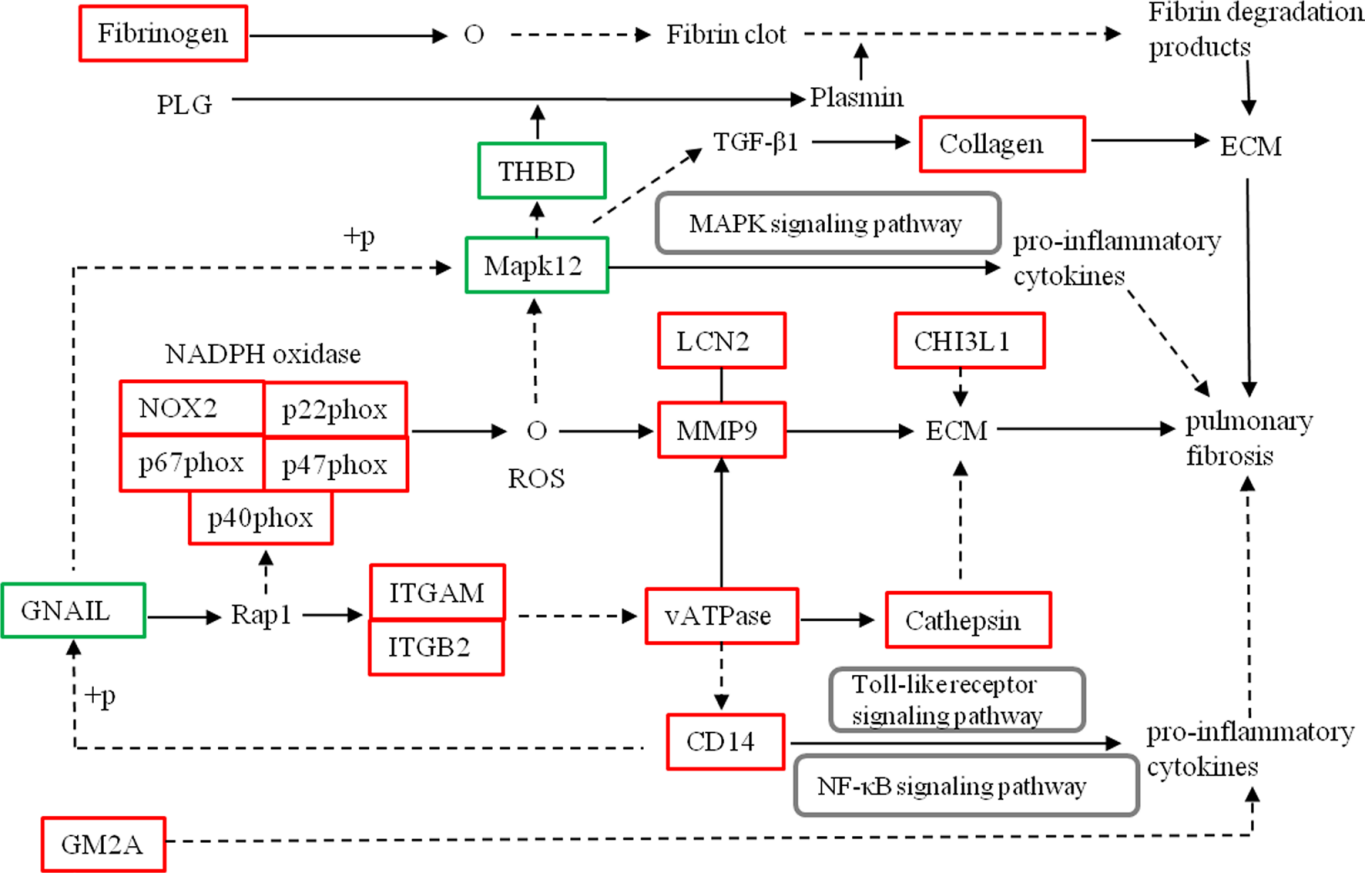
Inferred mechanisms of action of the cor-DEPs-DEGs genes with mainly enrichment pathways in the silica-treated rats. Red frame: upregulated; Green frame: downregulated;  : activate or leads to;  : indirect effect;  : complexes.  : combine; + p: phosphorylation; O: chemical molecules. For simplicity in presentation, only protein designations are shown

Chitinase 3-like 1 (CHI3L1) plays a protective role in tissue injury by ameliorating inflammation and cell death. It also has a profibrotic role in the repair phase by augmenting alternative macrophage activation, fibroblast proliferation and matrix deposition [36]. In bleomycin-induced IPF the expression of CHI3L1 was found to be decreased during the early injury phase and increased during fibroproliferative repair with increasing of matrix gene expression, collagen accumulation, a-SMA expression and secretion of TGF-β1. CHI3L1 can also interact with lung ECM to augment fibroblast proliferation and transformation to activated [37, 38]. CHI3L1 can also regulate liver cancer potentially by regulating the TGF-β signaling pathways with activating kinase to phosphorylate SMAD2 and SMAD3 [39]. In addition, Gm2a can cause transcription and immune responses to a range of inflammatory factors such as IL-6, TNF-α and TGF-β, which can promote fibrosis by activating NF-κB signal pathway [40, 41]. CD14, Toll-like receptor 2 and macrophage receptor with collagenous structure, could interact with cord factor to promote pro-inflammatory cytokine expression [42]. An analysis of pathways shows that these pathways are interrelated and converged at common points to produce an effect (Fig. 7).

Silicosis is an occupational disease characterized as inflammatory cells infiltration and progressive pulmonary fibrosis. In this study we analyzed the proteomic/transcriptomic profile in the lungs of rats on the 28th day after silica exposure when lung fibrosis has been formed according to our previous study. We could not provide the dynamic proteome changes occurring during silicosis. The function of these identified cor-DEG-DEP genes have not yet been explored. Further investigation is required to explore the roles of these identified cor-DEG-DEP genes in silicosis.

## Conclusions

In this study, we carried out a combined transcriptomic and proteomic analysis to study the molecular mechanisms underlying of pulmonary fibrosis in silica-exposed rats. Using joint analysis of transcriptomic and proteomic data we identified 250 DEPs/DEGs that mainly involve in phagosome, leukocyte transendothelial migration, complement and coagulation cascades and cell adhesion molecules (CAMs) pathways. These genes and their protein products could be critically important in initiation, progression and development of silicosis. Based on the results, we constructed the interaction map of these pathways to help understanding the molecular mechanism of silicosis (Fig. 7). Conclusively, this study provides important insights into molecular pathogenesis of silicosis and helpful information for more targeted prevention and treatment of the disease.

## Supplementary Information

**Additional file 1: Table S1.** The differentially expressed genes (DEGs) were expressed between the model and control groups.

**Additional file 2: Table S2.** The differentially expressed proteins (DEPs) were expressed between the model and control groups.

**Additional file 3: Table S3.** The cor-DEGs-DEPs genes with the same or opposite trend.

**Additional file 4: Table S4.** GO terms were highly enriched at both mRNA and protein levels.

**Additional file 5: Table S5.** KEGG terms were highly enriched at both mRNA and protein levels.

## Data Availability

The proteomic information including the raw data, peak list files etc., have been submitted to the PRIDE database with identifier PXD020625. The microarray gene expression data have been deposited in NCBI’s Gene Expression Omnibus (GEO; http://www.ncbi.nlm.nih.gov/geo/) under accession number GSE188520.

## Abbreviations

TMT: Tandem mass tags
GO: Gene Ontology
KEGG: Kyoto Encyclopedia of Genes
PRM: Parallel reaction monitoring
DEGs: Differentially expressed genes
DEPs: Differentially expressed proteins
CAMs: Cell adhesion molecules
ECM: Extracellular matrix
BP: Biological process
CC: Cellular component
MF: Molecular function;
GM2a: GM2 ganglioside activator
CHI3L1: Chitinase-3-like protein 1
LCN2: Neutrophil gelatinase-associated lipocalin
GNAI1: Guanine nucleotide-binding protein G(i) subunit alpha-1
NOXs: NADPH oxidase
IPF: Idiopathic pulmonary fibrosis
THBD: Thrombomodulin
MMPs: Matrix metalloproteinases
CHI3L1: Chitinase 3-like 1
